# Travel Burden and Timely Linkage to Care Among People Newly Diagnosed with HIV Infection in South Carolina from 2005 to 2020

**DOI:** 10.1007/s10461-024-04411-1

**Published:** 2024-06-17

**Authors:** Fanghui Shi, Jiajia Zhang, Peiyin Hung, Xiaowen Sun, Xueying Yang, Bankole Olatosi, Sharon Weissman, Xiaoming Li

**Affiliations:** 1 Arnold School of Public Health, South Carolina SmartState Center for Healthcare Quality, University of South Carolina, 915 Greene Street, Columbia, SC 29208 USA; 2 Department of Health Promotion, Education and Behavior, Arnold School of Public Health, University of South Carolina, Columbia, SC 29208 USA; 3 Department of Epidemiology and Biostatistics, Arnold School of Public Health, University of South Carolina, Columbia, SC 29208 USA; 4 Department of Health Services Policy and Management, Arnold School of Public Health, University of South Carolina, Columbia, SC 29208 USA; 5 Department of Internal Medicine, School of Medicine, University of South Carolina, Columbia, SC 29208 USA

**Keywords:** Travel burden, HIV, Linkage to care, Electronic health records

## Abstract

This retrospective study explored the association between travel burden and timely linkage to care (LTC) among people with HIV (PWH) in South Carolina. HIV care data were derived from statewide all-payer electronic health records, and timely LTC was defined as having at least one viral load or CD4 count record within 90 days after HIV diagnosis before the year 2015 and 30 days after 2015. Travel burden was measured by average driving time (in minutes) to any healthcare facility visited within six months before and one month after the initial HIV diagnosis. Multivariable logistic regression models with the least absolute shrinkage and selection operator were employed. From 2005 to 2020, 81.2% (3,547 out of 4,366) of PWH had timely LTC. Persons who had longer driving time (adjusted Odds Ratio (aOR): 0.37, 95% CI: 0.14–0.99), were male versus female (aOR: 0.73, 95% CI: 0.58–0.91), had more comorbidities (aOR: 0.73, 95% CI: 0.57–0.94), and lived in counties with a higher percentage of unemployed labor force (aOR: 0.21, 95% CI: 0.06–0.71) were less likely to have timely LTC. However, compared to those aged between 18 and 24 years old, those aged between 45 and 59 (aOR:1.47, 95% CI: 1.14–1.90) or older than 60 (aOR:1.71, 95% CI: 1.14–2.56) were more likely to have timely LTC. Concentrated and sustained interventions targeting underserved communities and the associated travel burden among newly diagnosed PWH who are younger, male, and have more comorbidities are needed to improve LTC and reduce health disparities.

**Supplementary Information:**

The online version contains supplementary material available at 10.1007/s10461-024-04411-1.

## Introduction

Timely linkage to care (LTC) ensures that persons newly diagnosed with HIV receive medical care promptly, essential for optimized HIV outcomes [1–3]. According to the Office of National AIDS Policy’s National HIV/AIDS Strategy for the United States, timely LTC involves receiving at least one CD4 or viral load test within three months (90 days) after HIV diagnosis by an HIV healthcare provider. However, in 2015, the objective of “linked to care” was changed from linkage within three months (90 days) to linkage within one month (30 days) [1–4]. In South Carolina (SC), 539 out of 729 (74%) persons diagnosed with HIV infection in 2021 were linked to care within one month, which was much lower than the SC Department of Health and Environmental Control (DHEC)’s goal of reaching 90% by December 31, 2024 [5]. Given the suboptimal prevalence of timely linkage to care in SC, it is imperative to understand the risk factors associated with delayed linkage to care to inform the development of targeted interventions and practical promotion strategies.

Travel burden is frequently cited as one of the top barriers to accessing care and achieving optimal health outcomes among people living with HIV (PWH), especially in rural states such as SC [6–11]. Proximity to care is closely related to HIV care continuum-related outcomes among PWH, including retention in care and viral suppression [12, 13]. Those living closer to care facilities or care providers were more likely to adhere to antiretroviral therapy (ART) and achieve viral suppression [14]. However, there are also some studies reporting that traveling long distances to healthcare was related to better HIV-related outcomes, such as increased retention in care. These anomalous results might be due to stigma and fear of HIV disclosure, and people chose to travel further for care to avoid being labeled as PWH in their own community [15]. In addition to mixed findings regarding the impact of travel burden on HIV-related outcomes, there is a paucity of evidence on earlier stages in the HIV care continuum, such as linkage to care, making it challenging to develop tailored interventions in the early stage for optimal HIV treatment. An ecologic study in Atlanta found that vehicle ownership at the ZIP-Code Tabulation Area (ZCTA)-level was significantly associated with LTC [16]. However, this study explored community-level instead of person-level transportation characteristics, and few studies account for individual and neighbourhood-level characteristics simultaneously, limiting the ability to interpret the association between transportation barriers and the HIV care continuum and identify potential mechanisms to improve HIV care [12]. 

By linking statewide population-based HIV electronic health records data to multiple physical and social contextual data (e.g., American Community Survey), this study investigates whether travel burden to usual healthcare providers was associated with timely LTC. We hypothesized that longer driving time to healthcare facilities was associated with timely LTC.

## Methods

### Population and Data Sources

The study population consists of all adult PWH (≥ 18 years old) diagnosed from January 2005 to November 2020 in SC with at least one visit to any healthcare providers (HIV or non-HIV) six months before and one month after the HIV diagnosis date. We restrict analyses to adults because of the heterogeneities in the experiences and challenges related to travel burden and HIV care access between adolescents and adults. Restricting analyses to adults allows for a more targeted investigation within the intended demographic and ensures that the findings are directly applicable to the adult population. Those without any usual healthcare visits during this time frame were excluded from the analysis because their travel burden information was not available, and we tried to avoid any assumption about their travel burden. Between 2005 and 2020, 20,092 persons newly diagnosed with HIV infection were identified in SC. After removing those without clinic visiting records within six months before and one month after HIV diagnosis (*n* = 15,314) and those below 18 years old at the year of HIV diagnosis (*n* = 412), 4,366 are eligible in the analysis. We extracted their deidentified information about age at diagnosis, gender, race/ethnicity, comorbidities, HIV transmission mode and residence from the SC statewide Enhanced HIV/AIDS Reporting System (eHARS), a database containing all HIV cases reported to the SC DHEC. The information about driving time to clinical visiting facilities was calculated by SC Revenue and Fiscal Affairs Office (RFA) based on the residential address and linked with the eHARS data. Detailed information regarding the database and the data linkage were described elsewhere [17]. We chose 2005 as the initial year of the current study because it marks the implementation of mandatory reporting of lab test results regarding CD4 count and viral load to SC eHARS in that year [17]. 

Individual-level data were then integrated with multiple publicly available county-level datasets (e.g., American Community Survey, Area Health Resources File, and County Health Rankings and Roadmaps Program) via residential county identifier, the Federal Information Procession Standards (FIPS) code. The human subject approval was obtained from the institutional review boards at the University of South Carolina and relevant SC state agencies.

### Outcome

According to the National HIV/AIDS Strategy for the United States: Updated to 2020 [1], timely LTC is defined as having at least one visit with an HIV medical provider within three months after being diagnosed with HIV before 2015. In 2015, the LTC target was updated to be within one month of HIV diagnosis. The Center for Disease Control and Prevention (CDC) surveillance data use the laboratory records of CD4 count or HIV RNA level (viral load) as the surrogate marker for the completion of a visit. Thus, the primary outcome of the current study, LTC, was a dummy variable indicating whether there was at least one CD4 or viral load record within 90 days after diagnosis before 2015 and within 30 days after HIV diagnosis after 2015. Individuals with the first CD4 or viral load record less than 90 days after HIV diagnosis before 2015 and 30 days after HIV diagnosis after 2015 were labelled as “1 = timely LTC”. Otherwise, they were labelled as “0 = delayed LTC”.

### Individual-level Predictors

The primary variable of interest was travel burden, measured by the average (mean) driving time (in minutes) from residence to healthcare facilities from 6-month pre-diagnosis to 1-month post-diagnosis [18]. Other individual-level predictors included demographic characteristics, comorbidities, HIV diagnosis year, and HIV transmission mode. Basic demographic characteristics were age at HIV diagnosis, gender (male/female), race/ethnicity (White, Black, Hispanic, and others), and residence (rural/urban). We used the 2013 USDA Rural-Urban Continuum Codes to differentiate the study population in 46 counties in SC into rural and urban residents, with codes 1–3 indicating urban residents (or metro) and codes 4–9 indicating rural residents (or non-metro). We calculated the adapted Charlson Comorbidity Index (CCI) score based on the ICD9/ICD10 codes to indicate the severity of comorbidities at the time of HIV diagnosis. The comorbidities included for calculating the CCI score and the correspondent ICD9/ICD10 codes defining these comorbidities are available in Supplemental Table 1. Considering the change in the definition of timely LTC in 2015 and the emergence of the COVID-19 pandemic in March 2020 in SC, the HIV diagnosis year was categorized into three groups in the analyses, including January 2005 to December 2015, January 2016 to March 2020, and April 2020 to December 2020. HIV transmission modes included heterosexual, injected drug use (IDU), men who have sex with men (MSM), IDU/MSM, and others/unknown.

### County-level Predictors

County-level variables associated with timely LTC were calculated or derived directly from multiple publicly available datasets [19]. Based on the concept of social determinants of health [20], county-level variables were conceptualized into five dimensions including: (1) economic stability (e.g., percentage of civilian labor force that is unemployed, median household income, Gini index of income inequality, and total civilian population for whom poverty status is determined); (2) education access & quality (e.g., percentage of population with less than high school education); (3) health care access and quality (e.g., percentage of population with no health insurance coverage, Health Professional Shortage Area code for shortage of primary care physicians and shortage of mental healthcare providers, and total number of mental health care providers per 100,000 population); (4) neighborhood & built environment (e.g., Black/White racial residential segregation index, White/non-White racial residential segregation index, percentage of housing units that are mobile homes, and percentage of housing units with no vehicle available); and (5) social & community context (e.g., percentage of population reporting Black or African American race, rural/urban status, population density, and percentage of households with same-sex unmarried partner). The detailed definition and data sources of each variable are given in Supplemental Table 2.

### Statistical Analysis

We used descriptive statistics (frequency/percentage for categorical variables and mean/standard deviation for continuous variables) to examine the socio-demographic characteristics of persons newly diagnosed with HIV by LTC status. Multivariable logistic regression models with the least absolute shrinkage and selection operator (LASSO) were employed to identify the most relevant variables associated with timely LTC from 2005 to 2020. The tuning parameter for LASSO was chosen based on the smallest misclassification error using 10-fold cross-validation with 20 iterations. A model with a smaller misclassification error and better conceptual meaning was chosen as the final model. Crude odds ratios (COR) and adjusted odds ratios (AOR) with 95% confidence intervals (CI) were presented. Statistically significant relationships were considered at *p* < 0.05. All statistical analyses were conducted in R 4.1.3.

## Results

### Descriptive Statistics

Of the 4,366 participants included in the analysis, 3,547 persons newly diagnosed with HIV (81.2% of 4,366) were timely LTC, 3,051(69.9%) were male, 3,279 (75%) were Black, and 2,953 (67.6%) had no comorbidities. The mean driving time for the participants was 17.7 min (Table 1), with an interquartile range of 5.8–21.6 min across all PWH.

**Table 1:** Socio-demographic distribution of timely LTC and delayed LTC among persons newly diagnosed with HIV in SC from 2005 to 2020

Characteristics	Total (*N* = 4,366)	Delayed LTC (*N* = 819)	Timely LTC (*N* = 3,547)	
**Age**				
18–24	988(22.6%)	210 (25.6%)	778 (21.9%)	
25–44	2066(47.3%)	398 (48.6%)	1668 (47.0%)	
45–59	1063(24.3%)	170 (20.8%)	893 (25.2%)	
>=60	249(5.7%)	41 (5.0%)	208 (5.9%)	
**Gender**				
Female	1315(30.1%)	208 (25.4%)	1107 (31.2%)	
Male	3051(69.9%)	611 (74.6%)	2440 (68.8%)	
**Race/ethnicity**				
Black	3279(75.1)	632 (77.2%)	2647 (74.6%)	
White	845(19.4%)	139 (17.0%)	706 (19.9%)	
Hispanic	163(3.7%)	30 (3.7%)	133 (3.7%)	
Other/Unknown	79(1.8%)	18 (2.2%)	61 (1.7%)	
**HIV transmission model**				
Heterosexual	937(21.5%)	137(16.7%)	800(22.6%)	
IDU^a^	174(4.0%)	23 (2.8%)	151 (4.3%)	
MSM^b^	1946(44.6%)	393 (48.0%)	1553 (43.8%)	
MSM/IDU	79(1.8%)	9 (1.1%)	70 (2.0%)	
Other/Unknown	1230(28.2%)	257 (31.4%)	973 (27.4%)	
**CCI Score**				
0	2953(67.6%)	516 (63.0%)	2437 (68.7%)	
1	899(20.6%)	177 (21.6%)	722 (20.4%)	
>= 2	514(11.8%)	126 (15.4%)	388 (10.9%)	
**Drive time [mean(SD)]**	17.7(20.5)	19.2 (24.4)	17.3 (19.5)	

^a^IDU: Injected drug use

^b^MSM: Men who have sex with men

### Multivariable Logistic Regression Models with LASSO Selection

The LASSO-based multivariable logistic regression model fits the relationship between driving time and timely LTC, and 17 (eight individual-level and nine county-level) out of the 35 characteristics were selected by LASSO. Persons newly diagnosed with HIV who reported longer driving time (aOR: 0.37, 95% CI: 0.14–0.99), were male vs. female (aOR: 0.73, 95% CI: 0.58–0.91), had two or more vs. no comorbidities (aOR: 0.73, 95% CI: 0.57–0.94) and lived in counties with a higher percentage of households with a same-sex unmarried partner (aOR: 0.37, 95% CI: 0.19–0.74) or with a higher unemployed rate (aOR: 0.33, 95% CI: 0.16–0.70) were less likely to have timely LTC. Persons newly diagnosed with HIV during the early COVID-19 pandemic (April 2020-November 2020) were much less likely to have timely LTC than their counterparts diagnosed in January 2005-December 2014 (aOR: 0.16, 95% CI: 0.05–0.54). However, compared to participants aged between 18 and 24, those aged between 45 and 59 (aOR: 1.47, 95% CI: 1.14–1.90) or those aged older than 60 (aOR: 1.71, 95% CI: 1.14–2.56) were more likely to have timely LTC. (Table 2)

**Table 2:** Factors with linkage to care in South Carolina: Lasso-based selectiona

	Crude OR (95% CI)	Adjusted OR (95% CI)	
***Individual-level variables***			
**Drive time**	0.32 (0.13, 0.82)*	0.37 (0.14, 0.99)*	
**Age**			
18–24	1	1	
25–44	1.13 (0.95, 1.98)	1.15 (0.94, 1.40)	
45–59	1.42 (1.13, 1.77)**	1.47 (1.14, 1.90)**	
>= 60	1.37 (0.95, 1.98)	1.71 (1.14, 2.56)**	
**Race/ethnicity**			
White	1	1	
Black	0.82 (0.67, 1.01)	0.82 (0.66, 1.02)	
Hispanic	0.87 (0.56, 1.35)	1.02 (0.65, 1.60)	
Other/Unknown	0.67 (0.38, 1.16)	0.85 (0.48, 1.52)	
**Gender**			
Female	1	1	
Male	0.75 (0.63, 0.89)**	0.73 (0.58, 0.91)**	
**Residence**			
Urban	1	1	
Rural	1.26 (1.03, 1.56)*	1.09 (0.82, 1.44)	
**HIV transmission model**			
Heterosexual	1	1	
Injected drug use (IDU)	1.12 (0.70, 1.81)	1.12 (0.68, 1.84)	
Men who have sex with men (MSM)	0.68(0.55, 0.84)*	0.97 (0.74, 1.28)	
MSM/IDU	1.33(0.65, 2.73)	1.68 (0.79, 3.58)	
Other/Unknown	0.65(0.52, 0.81)*	0.74 (0.58, 0.94)*	
**CCI Score**			
0	1	1	
1	0.86 (0.71, 1.04)	0.89 (0.73, 1.09)	
>= 2	0.65 (0.52, 0.81)***	0.73 (0.57, 0.94)*	
**HIV diagnose year**			
01/2005–12/2015	1	1	
01/2016–03/2020	0.35 (0.29, 0.41)**	0.23 (0.17, 0.32)**	
04/2020–12/2020	0.29 (0.09, 0.88)***	0.16 (0.05, 0.54)***	
***County-level variables***			
**Black %**	1.62 (1.13, 2.33)**	1.37 (0.68, 2.75)	
**Hispanic %**	0.36 (0.22, 0.60)**	1.17 (0.52, 2.65)	
**Disability %**	1.33 (0.89, 1.98)	2.15 (1.48, 1.04)*	
**Citizen %**	1.96 (1.12, 3.43)***	1.45 (0.68, 3.11)	
**Unemployed %**	6.18 (3.11, 12.29)***	0.21 (0.06, 0.71)*	
**Single child family %**	2.10 (1.15, 3.82)*	2.03 (0.55, 7.44)	
**Same-sex unmarried partner %**	1.69 (1.05, 2.71)*	0.37 (0.19, 0.74)**	
**Shortage of mental healthcare providers**	1.10 (0.85, 1.43)	1.30 (0.97, 1.74)	
**Uninsured %**	4.60 (2.79, 7.59)***	0.54 (0.25, 1.13)	

^a^All the individual-level factors listed in Table 1 and county-level factors listed in supplemental Table 2 were included in the Lasso-based selection

*p < 0.05, **p < 0.01, ***p < 0.001

## Discussion

This study is one of the first attempts to investigate the association of travel burden with timely LTC using an integrated dataset comprising EHR data and multiple public datasets. Our study contributes to the existing quantitative evidence on the relationship between travel burden and HIV LTC at the individual level. We found that a longer driving time between home and healthcare facilities was consistently associated with a lower likelihood of timely LTC, even after controlling for various individual and county-level characteristics. Additionally, younger persons, males, individuals with more comorbidities, and those living in counties with higher percentages of households with a same-sex unmarried partner or higher percentage of the unemployed labour force were less likely to be timely LTC. These subgroups with higher risks of delayed LTC found in the current study should be prioritized for targeted strategies to address their specific barriers and improve LTC.

When linking newly diagnosed PWH to care, driving time from the individual’s home and the clinic is important to consider. PWH having longer driving time from residence to healthcare facilities might face difficulties accessing timely HIV care. The delayed LTC deprives PWH of necessary healthcare, further increasing the risks of HIV transmission and comorbidities [2]. In turn, the associated comorbidities and HIV complications can worsen health in general. Yet, living in an underserved community where healthcare facilities are far away hinders their accessibility to engage in healthcare, potentially leading to detrimental outcomes [11, 12]. To break this cycle and improve HIV outcomes in a timely manner, policymakers and healthcare providers should consider strategies to mitigate such travel burdens for individuals living further away from treatment facilities. One pilot referral intervention study in Tanzania found that the provision of transportation allowance and community escort was related to higher registration at the HIV clinic within six months of diagnosis [21]. Besides providing transportation assistance for those living far away from healthcare facilities (e.g., a voucher for a vehicle ride share, referrals, and escort to the clinic), healthcare facilities could work with patients to schedule appointments at convenient times for patients and avoid appointments during rush hour [7, 21, 22]. 

However, some studies documented that PWH might travel farther than HIV-negative individuals for healthcare, which may be driven by fear of stigmatization after being recognized by their community members [23]. A systematic review of 66 studies across 15 countries found three studies reported a paradoxical beneficial impact of transportation barriers on HIV outcomes [24]. This review also found that studies with higher quality and larger numbers of participants were more likely to report that geographic and transportation-related barriers are related to worse outcomes throughout the HIV care continuum [24]. This may indicate that for most of the population, the travel burden remains a barrier to HIV care access despite potential stigma and fear of disclosure. For those willing to travel further and wait longer for care, healthcare facilities could work with patients to schedule appointments at convenient times for patients and avoid appointments during rush hour.

Our results indicate disparities in timely LTC by socio-demographic characteristics. Male persons, people with higher CCI scores, and those from counties with higher percentages of households with a same-sex unmarried partner or a higher percentage of the unemployed labour force were less likely to be timely LTC. The gender difference found in the current study is consistent with the U.S. Centers for Disease Control and Prevention (CDC)’s surveillance data, which showed the lowest percentage of timely LTC among male persons in SC in 2020 [25]. However, prior studies assessing gender differences in LTC have been conflicting, and they have shown lower LTC percentages in men [26], increased LTC percentages in men compared to women [27], and no gender differences [28]. The discordant results could be explained by varied populations and geographic locations [29]. Both crude and adjusted models in our study demonstrated reduced LTC in individuals with more comorbidities. This subgroup should be targeted to improve LTC with greater attention due to their higher risks of delayed LTC and more severe health outcomes than those without comorbidities.

Our findings also have some implications for resource allocation to improve timely HIV LTC at the county level. For example, a lower likelihood of timely linkage to care is associated with a higher percentage of same-sex unmarried partners and a higher percentage of the unemployed labour force. One explanation of the influence of the percentage of same-sex unmarried partners is the increased risk of not having health insurance or unmet medical needs among men or women in same-sex relationships compared to those in opposite-sex relationships [30]. Another potential explanation is the pervasive discrimination and rejection during healthcare-seeking experienced by same-sex partners due to their sexual orientation or gender identity [31–33]. Members of Lesbian, Gay, Bisexual, Transgender, Queer/Questioning, plus (LGBTQ+) tend to avoid and delay healthcare for fear of discriminatory interactions in healthcare, which lead to disparate health outcomes [33]. More resources should be allocated in communities at the most risk (e.g., a high proportion of same-sex unmarried couples) to enhance the collaboration among agencies to provide rapid LTC or expand peer advocates in clinics to assist clients with LTC [5]. During the pandemic, which started in March 2020 in the US, the travel burden became more concerning for timely LTC due to the city lockdown and cessation of public transportation [34–36]. To ensure continuing HIV care services, telehealth was recommended and implemented in numerous healthcare facilities [37, 38]. However, barriers to the utilization of telehealth during the pandemic included but were not limited to technological challenges, client/provider experience, and digital literacy [37]. Some studies found that participants prefer in-person services to telehealth, and the no-show rate for telehealth visits was high [39]. Still, telehealth may become a long-term mainstay in HIV care due to its convenience, less stigma, and added privacy.

Some limitations need to be acknowledged in the current study. First, we have no access to information about individual-level vehicle ownership and the transportation types among our study population, which significantly influences the travel burden individuals face. A patient living far away from health care facilities but having access to several cars and living in a wealthy suburb may not have the issue of travel burden. However, we accounted for the percentage of housing units with no vehicle available in each county, which we believe could partly address the variations in patients’ vehicle access. Second, our study used driving time as the proxy of travel burden, which may not necessarily equate to travel burden. Some other methods of estimating the travel burden to access HIV may be more accurate and need to be explored to compare the influence of different measures of travel burden. Third, the healthcare facilities measured in this study are any healthcare facilities that the patient visited within six months before and one month after the initial HIV diagnosis. We acknowledge that it might be more accurate to explore the travel burden to the HIV treatment facilities (e.g., Ryan White clinics). However, the outcome in the current study is timely linkage to care, defined as having at least one viral load/CD4 record within 30/90 days after diagnosis, and the viral load/CD4 tests can be done outside of HIV treatment facilities. Thus, we believe it is meaningful to explore the travel burden to any healthcare facility. Fourth, caution should be paid when generalizing findings of the current study since only individuals with clinical visiting information six months before and one month after HIV diagnosis were eligible for analysis. Our results can not be generalized to all persons newly diagnosed with HIV in SC, and we believe our study population might face higher risks of severe health outcomes if they have delayed LTC because of their worse health conditions indicated by clinical visits. Finally, SC is a predominantly rural state with a large percentage of residents living below the poverty line and with no robust public transportation system. Results may not be generalized to more urban and wealthy areas. Driving time and cost may vary depending on the community and environment.

Despite these limitations, our study has notable strengths. Prior studies investigating the impact of travel burden on LTC focused on community-level factors or were limited to a qualitative approach [16, 40]. However, we explored individual-level proximity to care, providing an additional level of accuracy and granularity.

## Conclusion

Efforts to end the HIV epidemic need to focus on the individual and socio-ecological factors that lead to disparities and risks in delayed LTC among persons newly diagnosed with HIV. The travel burden from residence to healthcare facilities was a significant structural barrier to timely LTC. Targeted and sustained interventions are needed for younger and male persons and those with more historical comorbidities. Promoting timely LTC in communities at the most risk (e.g., a high proportion of same-sex unmarried couples) with tailored interventions is warranted to improve linkage to care and HIV outcomes.

### Electronic Supplementary Material

Below is the link to the electronic supplementary material.

Supplementary Material 1

## Data Availability

The authors are prohibited from making individual-level data available publicly due to provisions in our data use agreements with state agencies/data providers, institutional policy, and ethical requirements. To facilitate research, we make access to such data available via approved data access requests through the data owners. The data is unavailable externally or for re-release due to prohibitions in data use agreements with our state agencies or other data providers. Institutional policies stipulate that all external requests for data access require collaboration with a USC researcher. For more information or to make a request, please contact (Bankole Olatosi, PhD): Olatosi@mailbox.sc.edu. The underlying analytical codes are available from the authors on request.

## Statements and Declarations

This work was supported by the National Institute of Allergy and Infectious Diseases of the National Institutes of Health under Award Numbers R56AI174896-01A1 and R01AI164947-01.
