# Supplementary material for: Travel Burden and Timely Linkage to Care Among People Newly Diagnosed with HIV Infection in South Carolina from 2005 to 2020
Source: AIDS Behav. 2024 Jun 17;28(8):2590–7. doi: 10.1007/s10461-024-04411-1 (PMC11286654; doi:10.1007/s10461-024-04411-1)
Supplement: Supplementary file 1 — Supplementary Material 1 [file 10461_2024_4411_MOESM1_ESM.docx]

| Supplemental Table 1 the comorbidities we included to calculate CCI and the corresponding ICD-9/ICD-10 code | | | |
| --- | --- | --- | --- |
| Comorbidities | ICD10 | ICD9 | Score |
| Myocardial infarction | I21. / I22. / I25.2 | 410. / 412. | 1 |
| Congestive heart failure | I09.9 / I11.0 / I13.0 / I13.2 / I 25.5 / I42.0 / I42.5 / I 42.6 / I42.7 / I42.8 / I42.9 / I43. / I50. / P29.0 | 428. / 398.91 / 402.01 / 402.03 / 402.11 / 402.91 / 404.01 / 404.03 / 404.11 / 404.13 / 404.91 / 404.93 / 425.4 / 425.5 / 425.6 / 425.7 / 425.8 / 425.9 | 1 |
| Peripheral vascular disease | I70. / I71. / I73.1 / I73.8 / I73.9 / I77.1 / I79.0 / I79.1 / I79.8 / K55.1 / K55.8 / Z95.8 / Z95.9 | 093.0 / 437.3 / 440. / 441. / 443.1 / 443.2 / 443.8 / 443.9 / 447.1 / 557.1 / V43.4 | 1 |
| Cerebrovascular disease | G45. / G46. / H34.0 / H34.1 / H34.2 / I60. / I61. / I62. / I63. / I64. / I65. / I66. /I67. / I68. | 431. / 432. / 433. / 434. / 435. / 436. / 437. / 438. / | 1 |
| Dementia | F01. / F02. / F03. / F04 / F05 / F06.1 / F06.8 / G13.2 / G13.8 / G30. / G31.0 / G31.1 / G31.2 / G91.4 / G94 / R41.81 / R54 | 290.0 / 290.1 /290.2 /290.3 / 290.4 / 294.0 / 294.1 / 294.2 / 294.8 / 331.0 / 331.1 / 331.2 / 331.7 / 797 | 1 |
| Chronic pulmonary disease | J40. / J41. / J42. / J43. / J44. / J45. / J46. / J47. / J60. / J61. / J62. / J63. / J64. / J65. / J66. / J67. / J68.4 / J70.1 / J70.3 | 490. / 491. / 492. / 493. / 494. / 495. / 496. / 500. / 501. / 502. / 503. / 504. / 505. / 506.4 / 508.1 / 508.8 | 1 |
| Rheumatic disease | M05. / M06. / M31.5 / M32. / M33. / M34. / M35.1 / M35.3 / M36.0 | 446.5 / 710.0 / 710.1 / 710.2 / 710.3 / 710.4 / 714. 0 / 714. 1 / 714. 2 / 714. 8 / 725. | 1 |
| Peptic ulcer disease | K25. / K26. / K27. / K28. | 531. / 532. / 533. / 534. | 1 |
| Mild liver disease | B18. / K70.0 / K70.1 / K70.2 / K70.3 / K70.9 / K71.3 / K71.4 / K71.5 / K71.7 / K73. / K74. / K76.0 / K76.2 / K76.3 / K76.4 / K76.8 / K76.9 / Z94.4 | 070.22 / 070.23 / 070.32 / 070.33 / 070.44 / 070.54 / 070.6 / 070.9 / 570. / 571. / 573.3 / 573.4 / 573.8 / 573.9 / V42.7 | 1 |
| Diabetes without chronic complication | E08 / E09 / E10 / E11 / E13 / E.0 / E.1 / E.6. / E.8. / E.9. | 250.8 / 250.9 / 249.0 / 249.1 / 249.2 / 249.3 / 249.9 | 1 |
| Renal (mild or moderate) | I12.9 / I13.0 / I13.1 / N03. / N05. / N18.1 / N18.2 / N18.3 / N18.4 / N18.9 / Z94.0 | 430.00 / 430.10 / 430.90 / 404.00 / 404.01 / 404.10 / 404.11 / 404.90 / 404.91 / 582. / 583. / 585.1 / 585.2 / 585.3 / 585.4 / 585.9 / V42.0 | 2 |
| Diabetes with chronic complication | E08 / E09 / E10 / E11 / E13 / E.2. / E.3. / E.4. / E.5. | 250.4 / 250.5 / 250.6 / 250.7 | 2 |
| Hemiplegia or paraplegia | G04.1 / G11.4 / G80.0 / G80.1 / G80.2 / G81. / G82. / G83. | 334.1 / 342. / 343. / 344. | 2 |
| Any malignancy, including lymphoma and leukemia, except malignant nonmelanoma neoplasm of skin | C0. / C1. / C2. / C30. / C31. / C32. / C33. / C34. / C37. / C38. / C39. / C40. / C41. / C43. / C45. / C46. / C47. / C48. / C49. / C50. / C51. / C52. / C53. / C54. / C54. / C56. / C57. / C58. / C59. / C60. / C61. / C62. / C63. / C76. / C80.1 / C81. / C82. / C83. / C84. / C85. / C88. / C9. | 14. / 15. / 16. / 170. / 171. / 172. / 174. / 175. / 176. / 179. / 18. / 190. / 191. / 192. / 193. / 194. / 195. / 199.1 / 200. / 201. / 202. / 203. / 204. / 205. / 206. / 207. / 208. / 238.6 | 2 |
| Moderate or severe liver disease | I85.0 / I86.4 / K70.4 / K71.1 / K72.1 / K72.9 / K76.5 / K76.6 / K76.7 | 456.0 / 456.1 / 456.2 / 572.2 / 572.3 / 572.4 / 572.8 | 3 |
| Renal (severe) | I12.0 / I13.11 / I13.2 / N18.5 / N19. / N25.0 / Z49. / Z99.2 | 403.01 / 403.11 / 403.91 / 404.02 / 404.02 / 404.03 / 404.12 / 404.13 / 404.92 / 404.93 / 585.5 / 585.6 / 586. / 588.0 / V45.11 / V45.12 / V56.0 / V56.1 / V56.2 / V56.31 / V56.32 / V56.8 | 2 |
| Metastatic solid tumor | C77. / C78. / C79. / C80.0 / C80.2 | 196. / 197. / 198. / 199.0 | 6 |

| Supplemental Table 2 Definition and data sources of 27 county-level characteristics | | |
| --- | --- | --- |
| Characteristics | Definition | Data sources |
| **Social & community context** |  |  |
| Black % | Percentage of population reporting Black or African American race alone | American Community Survey (ACS) |
| Hispanic % | Percentage of population reporting Hispanic ethnicity | ACS |
| Total population | Total weighted population | ACS |
| <=17 % | Percentage of population between ages 0-17 | ACS |
| >= 65 % | Percentage of population ages 65 and over | ACS |
| disability % | Percentage of population with a disability | ACS |
| single child family % | Percentage of families with children that are single-parent families | ACS |
| Population density | Population density (County) | ACS |
| Same-sex unmarried partner % | Percentage of households with same-sex unmarried partner | ACS |
| Citizen % | Percentage of population who are citizens (ages 18 and over) | ACS |
| Non-citizen % | Percentage of population who are not U.S. citizens and entered U.S. since 2010 | ACS |
| **Economic stability** |  |  |
| Poverty % | Total civilian population for whom poverty status is determined (ages 18 and over) | ACS |
| Unemployed % | Percentage of civilian labor force that is unemployed (ages 16 and over) | ACS |
| Median income | Median household income (dollars, inflation-adjusted to data file year) | ACS |
| Gini index | Gini index of income inequality | ACS |
| **Education access & quality** |  |  |
| Less than high school % | Percentage of population with less than high school education (ages 25 and over) | ACS |
| **Neighborhood &built environment** |  |  |
| Black/White residential segregation index | Segregation Index (higher values indicate greater residential segregation between Black and White residents) | County Health Rankings (CHR) |
| White/non-White residential segregation index | Segregation Index (higher values indicate greater residential segregation between non-White and White residents) | CHR |
| Isolation index | The extent to which minority members are exposed only to one another, and is computed as the minority-weighted average of the minority proportion in each area | Calculated |
| Dissimilarity index | The percentage of a group's population that would have to change residence for each census tract to have the same percentage of that group as the county area overall. The index ranges from 0.0 (complete integration) to 1.0 (complete segregation). | Calculated |
| Mobile homes % | Percentage of housing units that are mobile homes | ACS |
| No access to vehicle % | Percentage of housing units with no vehicle available | ACS |
| **Health care access & quality** |  |  |
| Mental health care providers per 100,000 | Total number of mental health care providers per 100,000 population | CHR |
| Shortage of primary care physicians | Health Professional Shortage Area (HPSA) code-shortage of primary care physicians | Area Health Resources file (AHRF) |
| Shortage of mental healthcare providers | Health Professional Shortage Area (HPSA) code-shortage of mental healthcare providers | AHRF |
| Uninsured % | Percentage of population with no health insurance coverage | ACS |
| Uninsured (<=64) % | Percentage of population with no health insurance (ages 64 and below) | ACS |
